# Computational understanding of non-coding RNA pairwise interactions

**DOI:** 10.3389/frai.2026.1749205

**Published:** 2026-02-18

**Authors:** Marco Nicolini, Federico Stacchietti, Elena Casiraghi, Giorgio Valentini

**Affiliations:** 1 AnacletoLab, Dipartimento di Informatica, Universitá degli Studi di Milano, Milan, Italy; 2 European Lab for Learning and Intelligent Systems (ELLIS), Milan, Italy; 3 Environmental Genomics and Systems Biology Division, Lawrence Berkeley National Laboratory, Berkeley, CA, United States; 4 Department of Computer Science, Aalto University, Espoo, Finland

**Keywords:** ncRNA-ncRNA interaction, deep learning, fine-tuning, artificial intelligence, machine learning, non-coding RNA, large language models

## Abstract

Non-coding RNAs (ncRNAs) govern a vast network of regulatory interactions within the cells, yet their pairwise relationships remain largely uncharted due to the complexity of RNA structure and the limits of current experimental methods. We present *CUPID* (Computational Understanding of Pairwise Interactions in ncRNA Data), a deep learning framework that predicts ncRNA-ncRNA interactions directly from primary sequence information. *CUPID* uses embeddings from a pre-trained RNA language model combined with a feed-forward classifier to identify patterns linked to molecular pairing. This approach avoids reliance on thermodynamic models or manual feature design and, unlike previously proposed models, is able to generalize across different types of ncRNAs, including long non-coding, circular, micro-, and small nuclear RNAs. By learning the hidden rules that govern RNA recognition, *CUPID* provides a scalable tool for exploring ncRNA interaction networks and advancing our understanding of RNA-based regulation.

## 1 Introduction

Understanding RNA-RNA interactions is critical for deciphering the regulatory circuits that orchestrate gene expression, RNA processing, and signal transduction. Non-coding RNAs (ncRNAs), despite lacking protein-coding potential, play pivotal roles in these processes (Ali et al., 2021). However, experimental mapping of ncRNA interactions remains challenging due to the limitations of existing experimental and computational techniques (Lorenzi et al., 2021).

Methods such as Minimum Free Energy (MFE) calculations and accessibility-based models have been usually applied to predict RNA-RNA interactions. Tools like IntaRNA (Mann et al., 2017) estimate the interaction energy as Δ*G*_total_ = Δ*G*_duplex_+Δ*G*_accessibility_, where the first term quantifies the energy released upon hybridization, and the second accounts for the cost of rendering binding regions accessible. Benchmark studies have demonstrated that accessibility-based algorithms can effectively differentiate native interactions from background noise (Umu and Gardner, 2017), yet these approaches rely on predefined parameters and simplified energy models. In parallel, experimental techniques such as RNA Antisense Purification (RAP-RNA) offer validation but remain limited by their high cost and labor intensity (Engreitz et al., 2014).

Advances in machine learning and graph-based modeling for biological data, including recent work on explainability and diffusion-based attention mechanisms, have motivated a surge of learning-driven approaches for predicting interactions across diverse molecular systems (Gliozzo et al., 2025; Cetin and Sefer, 2025; Sefer, 2025).

Machine learning methods, such as convolutional neural networks, deep forests and graph neural networks (Alipanahi et al., 2015; Tian et al., 2021; Wei et al., 2022) have been applied to RNA-protein interaction prediction, while graph-based approaches embed heterogeneous networks of ncRNAs and diseases using multigraph contrastive learning (Sun et al., 2025) or apply random-walk based graph representation learning techniques to predict non coding RNA interactions (Torgano et al., 2025).

While effective, these methods often rely on predefined feature extraction, graph structures, or supervised training, limiting their adaptability to novel ncRNA sequences.

In contrast, LLMs can directly learn from large corpora of proteins or RNA data (Valentini et al., 2023; Zhao et al., 2024; Shen et al., 2024; Nicolini et al., 2025a), capturing intricate interaction motifs beyond predefined energy models or graph-based constraints. Unlike thermodynamic models, which impose simplifying assumptions, LLMs infer interaction likelihoods from latent structural patterns, offering a flexible, data-driven approach. In particular, transformer-based foundation models can generate biologically meaningful representations directly from raw sequences, by exploiting large RNA sequence corpora (Sapoval et al., 2022; Chen et al., 2022; Yu et al., 2024). More in general several deep learning methods have been proposed to predict specific ncRNA interactions, using rna2vec pre-training and deep feature mining (Yu et al., 2022) or conditional random fields and graph convolutional networks (Wang et al., 2022), heterogeneous graph neural networks (Li et al., 2025) and convolutional neural networks combined with a Transformer Encoder (Yang et al., 2025) for the prediction of miRNA-lncRNA interactions.

We also recently proposed a deep neural network trained on embedded representations of a subset of ncRNAs obtained from the RNA-FM language model (Shen et al., 2024), achieving state-of-the-art results for predicting miRNA interactions with other ncRNA molecules (Nicolini et al., 2025b). However, our proposed model, like other models recently proposed in the literature (Li et al., 2025; Yang et al., 2025), is only able to predict specific ncRNA interactions (e.g., interactions with miRNAs). Furthermore, due to limitations on the maximum allowed sequence length of the underlying RNA-FM transformer, it can only process sequences shorter than approximately 1,000 nucleotides, thus limiting the model's application to relatively long ncRNAs (e.g., lncRNAs).

To overcome these limitations, we propose a novel Transformed-based deep learning model, that, differently from previous models proposed in literature, is able to predict a large range of ncRNA interactions, including long non-coding RNA (lncRNA), circular RNA (circRNA), microRNA (miRNA), small nuclear RNA (snRNA), small nucleolar RNA (snoRNA), Small Cajal body-specific RNAs (scaRNAs), small cytoplasmic RNAs (scRNA), and other types of ncRNAs. Moreover, by adopting GenerRNA (Zhao et al., 2024) to encode RNA sequences, our model can process full-length ncRNA sequences (up to 4,096 nucleotides) without truncation, thus significantly enlarging the set of ncRNAs that can be processed by the model.

We hypothesize that LLM-based contextual embeddings provide a rich representation for ncRNA interaction prediction, circumventing the limitations of manual feature engineering or predefined structural graphs. We reasoned that GenerRNA (Zhao et al., 2024), pretrained on a large corpus of ncRNA sequences using a masked language modeling objective, can capture long-range interactions of ncRNA molecules, thus facilitating downstream tasks such as ncRNA interaction prediction.

Our *CUPID* model (Computational Understanding of Pairwise Interactions in ncRNA Data), predicts ncRNA interactions using only sequence information. *CUPID* extracts embeddings from a pre-trained ncRNA language model and feeds a dense feed-forward neural network (FFNN) to automatically learn intricate sequence interaction features. This design circumvents the need for explicit thermodynamic parameterization and manually engineered features, offering a scalable and efficient alternative for uncovering novel regulatory interactions (Fabbri et al., 2019).

## 2 Methods

### 2.1 Dataset

Our dataset comprises a subset of multispecies ncRNA interaction pairs from RNA-KG (Cavalleri et al., 2024).^1^

The RNA-KG integrates physical and functional interactions between different types of ncRNAs, and their relationships with other biomolecules (genes and proteins) and chemicals, as well as with biomedical concepts coded in the Gene Ontology (Aleksander et al., 2023), the Human Phenotype Ontology (Gargano et al., 2024), Mondo (Vasilevsky et al., 2025), and other bio-medical ontologies related to the “RNA world.”

**Figure 1:**
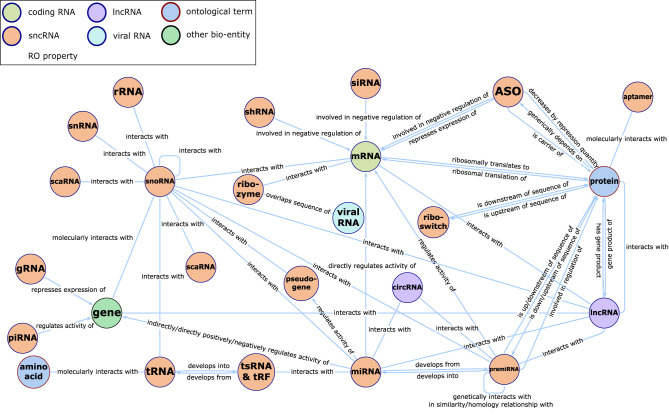
Simplified representation of the RNA-KG meta-graph, focused on ncRNAs and their interactions.

In particular, we extracted RNA–RNA edges from RNA-KG by selecting only relations annotated as interacts-with. In RNA-KG, interacts-with denotes experimentally supported *physical* RNA–RNA interactions, and we therefore excluded other relation types encoding functional associations (e.g., regulatory links, co-expression, or disease associations). The interacts-with edges integrated in RNA-KG originate from multiple underlying curated interaction databases. Figure 1 presents an overview of the main RNA entities and their relationships available in the the RNA-KG. Readers may refer to the RNA-KG reference (Cavalleri et al., 2024) for the complete list of contributing sources and evidence provenance.

We filtered the dataset to retain only sequences that fit within the GenerRNA (Zhao et al., 2024)'s token limit (approximately 4,096 nucleotides), since Byte Pair Encoding (BPE) compresses raw nucleotide sequences, allowing longer sequences to fit within the model's constraints. After applying this length filter, the dataset contains:

- 101, 088 interaction pairs (down from an initial 130, 310 pairs).
- 11, 212 unique sequences (selected from 19, 624 potential sequences) belonging to 9 different RNA molecule types: long non-coding RNA (lncRNA), circular RNA (circRNA), microRNA (miRNA), small nuclear RNA (snRNA), small nucleolar RNA (snoRNA), Small Cajal body-specific RNAs (scaRNAs), small cytoplasmic RNAs (scRNA), not (better) classified non coding RNA molecules (ncRNA), and pseudo RNA.^2^

In the following, we denote the set of length-filtered molecules as


$$S=\left\{s_{i}\right\},\;i=1,\ldots ,|S|,$$

where the type of each molecule s∈S is given by ϕ(*s*), i.e. ϕ:S→T represents a mapping of a ncRNA sequence s∈S to its ncRNA type T, e.g. miRNA, lncRNA or any other ncRNA type.

The identity of an interaction pair is solely determined by its constituent molecules, regardless of order; that is,


$$\left(s_{i},s_{j}\right)=\left(s_{j},s_{i}\right).$$

The type of an interaction (*s*_*i*_, *s*_*j*_) with *s*_*i*_≠*s*_*j*_ and $s_{i},s_{j}\in S$ is determined by the types of the ncRNA *s*_*i*_ and *s*_*j*_ theirselves, regardless of their order:


$$\left(\phi \left(s_{i}\right),\phi \left(s_{j}\right)\right)=\left(\phi \left(s_{j}\right),\phi \left(s_{i}\right)\right)$$

**Figure 2:**
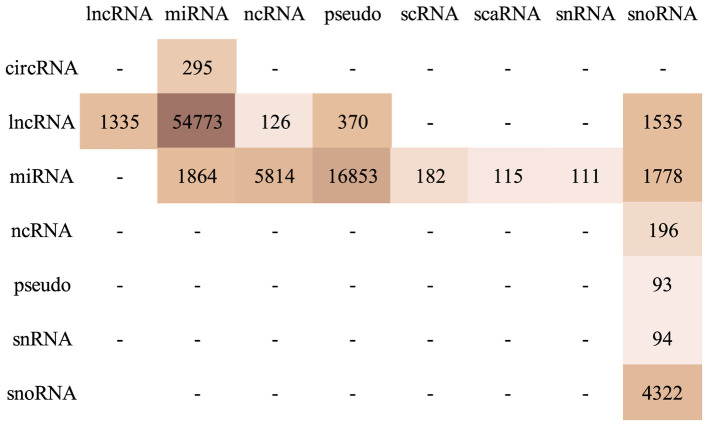
Distribution of ncRNA interactions pairs in the filtered interaction set. Rows: first (left) molecule type; Columns: right molecule type.

For instance, possible types of ncRNA interactions are miRNA-lncRNA or miRNA-circRNA. Assuming that interacting ncRNA pairs of different types exhibit distinct specificities that the model should learn, we reasoned that types with negligible sample sizes might introduce noise rather than valuable information. Therefore, the set of interaction pairs used in this work is obtained by further filtering the dataset of interacting pairs to remove interacting pair types represented by fewer than 100 samples, resulting in 10,644 unique sequences composing 99,841 interacting pairs. Figure 2 shows the distribution of the different types of ncRNA interactions.

### 2.2 Data augmentation

To address the issues due to the limited cardinality of the available training data, especially for specific types of ncRNA interactions (e.g., snRNA-miRNA or miRNA-circRNA), we employ a data augmentation strategy that effectively increases the dataset size by a factor of 4. For each original training instance represented as a pair of interacting ncRNA (*s*_*i*_, *s*_*j*_) we generate three additional augmented instances:

1. Molecule Order Reversal: Swap the order of the molecules: (*s*_*j*_, *s*_*i*_).
2. Sequence Flipping: Reverse the nucleotide order in both molecules (denoted by the superscript *F*): $\left(s_{i}^{F},s_{j}^{F}\right)$.
3. Combined Augmentation: Reverse both the molecule order and the nucleotide sequences: $\left(s_{j}^{F},s_{i}^{F}\right)$.

Thus, if the original dataset contains *N* instances, the augmented dataset becomes: *N*_aug_ = 4*N* (Supplementary Figure S1). This augmentation introduces invariance to both the order and orientation of sequences, thereby enabling the model to better capture the underlying biological patterns and improving its robustness against input variability.

In order to avoid leakage between training and test sets, data augmentation is performed after splitting the dataset.

### 2.3 Negative examples generation

In our dataset, only positive non-coding RNA-RNA interactions are explicitly provided, and they occur with varying frequencies.

To effectively train *CUPID* , we generated negative examples for each interaction pair type by matching the frequency distribution of the positive interactions. Specifically, negative examples were generated under the assumption that any pair of ncRNA sequences drawn from the set of unique sequences that is not observed as a positive interaction constitutes a possible negative instance.

Let $S=\left\{s_{1},s_{2},\ldots ,s_{N}\right\}$ be the set of unique ncRNA sequences present in the dataset. Denote by


$$P=\left\{\left(s_{i},s_{j}\right)|s_{i},s_{j}\in S\text{interact}\right\}$$

the set of all positive ncRNA-ncRNA interactions. Then, the set of all possible ncRNA pairs is given by S×S (excluding self-interactions).

The set of *potential negatives* is defined as:


$$N_{\text{potential}}=\left\{\left(s_{i},s_{j}\right)\in S\times S|s_{i}\neq s_{j}\right\}\backslash P.$$

*Negative sampling procedure* To generate the negative samples for each interacting pair type, we corrupt its tuples. In other words, given a positive pair (*s*_*i*_, *s*_*j*_) with type (ϕ(*s*_*i*_), ϕ(*s*_*j*_)), we keep the first molecule *s*_*i*_ fixed and sample $s^{\prime }\in S$ such that:


$$s^{\prime }\neq s_{i},\;\phi \left(s^{\prime }\right)=\phi \left(s_{j}\right),\;\left(s_{i},s^{\prime }\right)\notin P$$

In this way we avoid generating negatives between ncRNA types that never interact (e.g., scaRNA and lncRNA).

Because we generate negatives for each positive pair (*s*_*i*_, *s*_*j*_) by corrupting the right molecule while keeping the same type pair (ϕ(*s*_*i*_), ϕ(*s*_*j*_)), the negative set preserves the interaction *type-pair* distribution of the positives in expectation (and approximately in practice, up to rejection of candidates already present as positives or previously sampled negatives).

For each positive edge, we selected *n* negative edges, in order to control the imbalance between positive and negative edges in the testing phase (we set *n* = 20 in our experiments).

*Negative sampling algorithm* The negative sampling algorithm is detailed in Algorithm 1. In our implementation, we set *n* = 20. Note that, since the condition at line 5 of the algorithm cannot be always guaranteed, it is likely that the number of negatives *n* ≤ 20. In our experiments we set *n* = 20.

Algorithm 1 Negative sampling algorithm. 
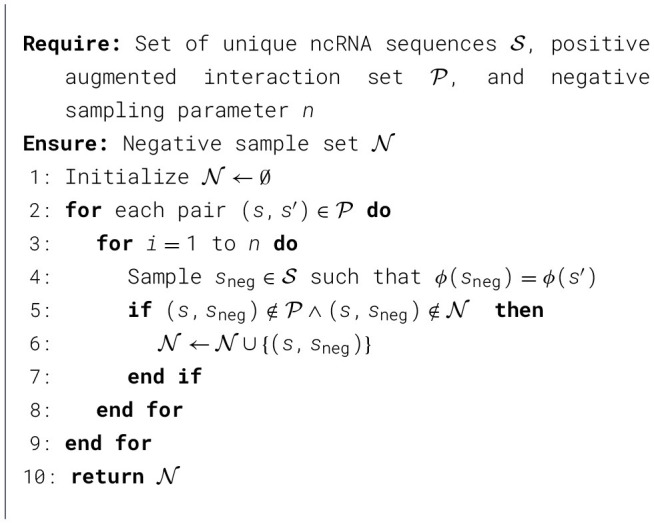

### 2.4 Model architecture

#### 2.4.1 The overall *CUPID* architecture

**Figure 3:**
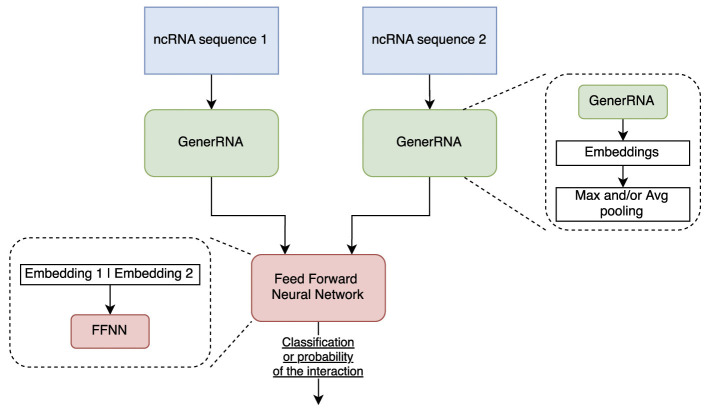
High-level *CUPID* architecture schema.

Our model follows a two-stage pipeline, as illustrated in Figure 3. It first extracts ncRNA sequence embeddings using a pre-trained ncRNA Language Model (GenerRNA; Zhao et al., 2024) and then processes these embeddings through a Feed-Forward Neural Network (FFNN) to predict interaction probabilities.

The GenerRNA architecture mimics the GPT-2-medium model (Radford et al., 2019), and is composed of 24 stacked transformer-decoder layers, each incorporating a self-attention mechanism that models pairwise interactions among all positions in its input sequence. GenerRNA uses a context window of 1,024 tokens, corresponding to input sequences with a length of approximately 4,096 nucleotides coded through byte pair encoding (Sennrich et al., 2016). Note that this maximum length permits the encoding of large RNA molecules. This decoder-only Transformer architecture operates in an autoregressive manner, predicting the subsequent token given the previous ones. Both the input and output of the model are represented as tokens, which are encoded and decoded by a trained tokenizer using byte pair encoding. A special token (EOS) is used to delimit sequences, indicating the start and end of each sequence.

**Figure 4:**
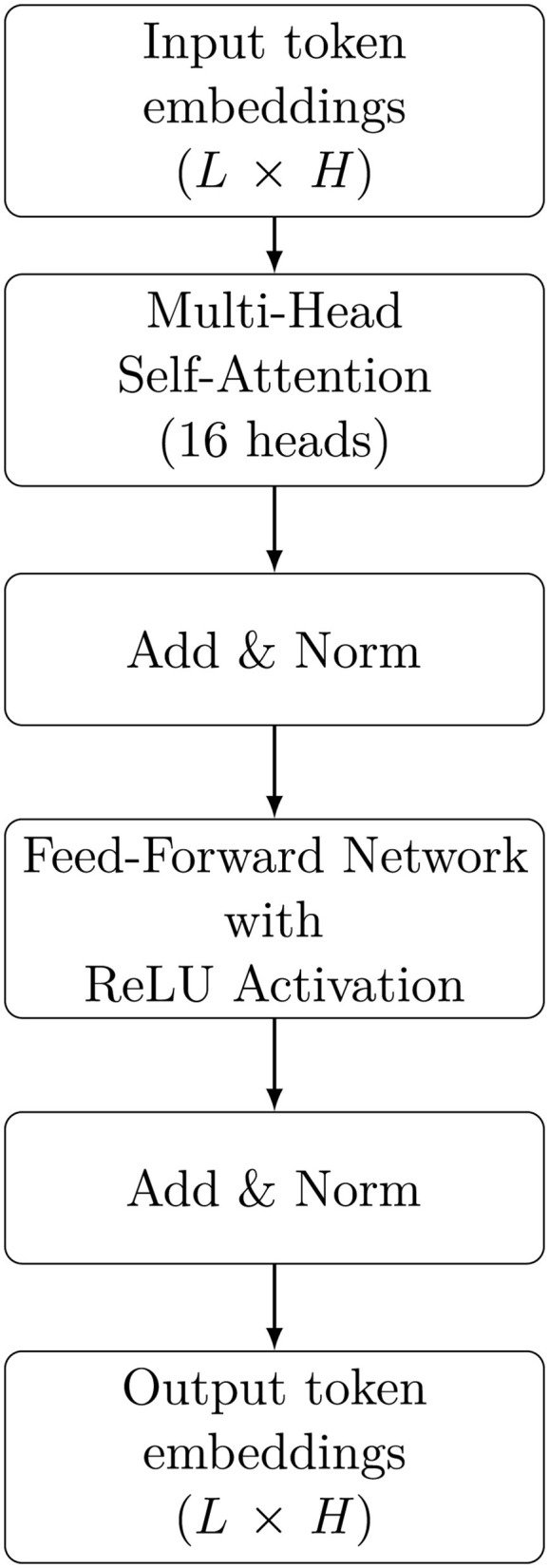
High-level architecture of a GenerRNA block.

Each transformer block is fed with a input of size *L*×*H*, thus allowing to process RNA sequences having up to *L* tokens, each one represented through a *H*-dimensional real vector, with *L* = *H* = 1, 024, and outputs a latent representation with the same dimensionality for each input token. For each input sequence, the block employs a multi-head self-attention mechanism with 16 attention heads. This is followed by an “Add & Norm” sub-block, which applies residual addition and layer normalization. Subsequently, a feed-forward sub-layer expands the hidden states from 1,024 to 4,096 dimensions, applies a non-linear activation (ReLU), and then projects them back to 1,024 dimensions. Another “Add & Norm” sub-block is applied after the feed-forward network, and finally, the block produces an output matrix **X**∈ℝ^*L*×*H*^. A schematic diagram of this block is reported in the Figure 4.

#### 2.4.2 Pooling techniques

The *i*^*th*^ row of matrix **X** is a latent representation ${\text{x}}_{i}\in {\mathbb{R}}^{1024}$ of the *i*^*th*^ token. To obtain a fixed-length embedding for the entire sequence, we tested two types of pooling over the sequence (i.e., across the *H* tokens), as well as their concatenation:

- Average (*Avg*) Pooling: obtained as the mean of the embeddings of all the tokens: ${\text{e}}_{\text{avg}}=\frac{1}{L}\overset{L}{\underset{i=1}{\sum }}{\text{x}}_{i}.$
- Maximum (*Max*) Pooling: Compute the element-wise maximum over all token embeddings: ${\text{e}}_{\text{max}}=\underset{i\in 1\ldots L}{cmax}\left[x_{i1},x_{i2},\ldots x_{iH}\right]$, where *cmax* is the columnwise *max* operator, and *x*_*ij*_ are the elements of the **X** embedding matrix.
- Concatenation of [*Avg, Max*]: Combine both pooled representations into a single embedding vector: $\text{e}=\left[{\text{e}}_{\text{avg}};{\text{e}}_{\text{max}}\right]\in {\mathbb{R}}^{2,048}.$

**Figure 5:**
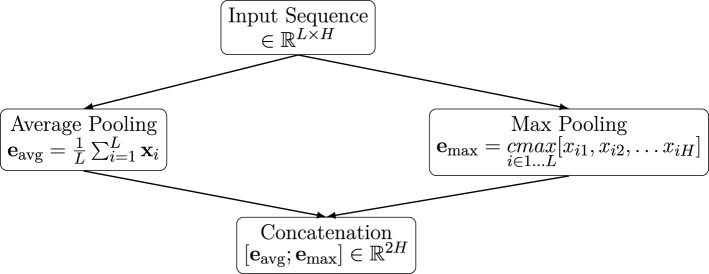
Pooling embedding strategies.

These pooling strategies are schematically depicted in Figure 5.

The embedded representation of a candidate interacting ncRNA pair is composed by the concatenation of the embeddings of the two interacting molecules.

#### 2.4.3 The classification unit

To predict the interaction we used the pooled embeddings of the RNA sequences as input to a dense Feed Forward Neural Network (FFNN), having the following architecture:

- **Input layer dimension:** 1,024 for Avg and Max-pooling embedding strategies, 2,048 when the embedding of the input molecule is obtained by concatenating the embeddings obtained by AVG and Max pooling,
- **Hidden layers:** 4 hidden layers with 1,024 neurons each and ReLU activation function,
- **Output layer:** 1 neuron with sigmoid activation function.

To train the network we applied the following hyper-parameters:*Learning Rate:* η = 5 × 10^−4^ with a linear warm-up phase of 4 epochs, followed by cosine decay. *Epochs:* 50 epochs with early stopping (patience of 10 epochs). The model with the best validation loss is selected (e.g., if the lowest validation loss is observed at epoch 35, then early stopping is triggered at epoch 45, and the model from epoch 35 is used).*Batch Size:* 512. *Dropout Rate:* 0.2. *Optimizer:* Adam. *Loss Function:* Binary Cross-Entropy.

Training and validation loss curves were monitored over epochs to assess model convergence and to avoid potential overfitting by early stopping.

#### 2.4.4 Mini-batch balancing

Due to the imbalance in our dataset we adopted a training strategy designed to prevent the model from learning predominantly from the negatives. To address this, we constructed mini-batches that contain a controlled mix of positive and negative examples. Recall that our training set is composed of the set of positive interaction pairs, $P,|P|=N_{+}$, and the set of negative interaction pairs N, with $|N|=N_{-}=nN_{+}$, as detailed in Section 2.3. Each mini-batch *B* of size *m* is formed by randomly selecting *m*_*p*_ positive examples (using a uniform distribution with replacement) and *m*_*n*_ negative examples (using a uniform distribution without replacement). The ratio of negatives within each mini-batch is defined by


$$r=\frac{m_{n}}{m_{n}+m_{p}},\text{ with }m_{n}+m_{p}=m.$$

Here, *r* can vary between 0 and 1. A value of *r* = 0.7 implies that 70% of the mini-batch consists of negatives. The choice to sample positives with replacement is driven by their limited number, ensuring sufficient representation even in large batches, whereas sampling negatives without replacement allows for a broader coverage of these more abundant examples.

### 2.5 Experimental evaluation

#### 2.5.1 Data preparation and splitting

In all our experiments the negative examples were sampled according to the relative frequency of the interacting pair types according to the procedure described in Section 2.3 using a negative:positive ratio equal to 20:1.

The dataset was partitioned into stratified training and test sets (train:test ratio = 90:10). The training set was further split into a stratified set for training (80% of interaction pairs) and the remaining 20% for validation. The validation set was used for early stopping and for tuning the classification threshold via maximization of the Matthews correlation coefficient (MCC; Matthews, 1975) on the validation data.

#### 2.5.2 Evaluation metrics

To comprehensively assess model's performance, we computed a range of evaluation metrics, encompassing both threshold-dependent and threshold-independent measures. Specifically, we first evaluated standard binary classification metrics, including accuracy, balanced accuracy (to account for class imbalance), precision, recall, F1 score, AUROC (Area Under the Receiver Operating Characteristic Curve), and AUPRC (Area Under the Precision-Recall Curve). In addition to these overall metrics, we conducted a stratified analysis based on interacting pair types, computing the aforementioned measures separately for each pair type.

Let *y*_*i*_∈{0, 1} be the ground-truth label and ${\hat{p}}_{i}\in \left[0,1\right]$ the predicted probability for sample *i*. Given a decision threshold *t*, we define ${ŷ}_{i}=1\;\Leftrightarrow \;\left[{\hat{p}}_{i}\geq t\right]$ and the confusion matrix counts:


$$\begin{array}{l} \text{TP}=\underset{i}{\sum }\text{I}\left[y_{i}=1\wedge {ŷ}_{i}=1\right],\\ \text{TN}=\underset{i}{\sum }\text{I}\left[y_{i}=0\wedge {ŷ}_{i}=0\right],\\ \text{FP}=\underset{i}{\sum }\text{I}\left[y_{i}=0\wedge {ŷ}_{i}=1\right],\\ \text{FN}=\underset{i}{\sum }\text{I}\left[y_{i}=1\wedge {ŷ}_{i}=0\right]. \end{array}$$

Threshold-dependent metrics are then computed as:


$$\begin{array}{c} \text{Accuracy(rate of correctly predicted instances)}\\ =\frac{\text{TP}+\text{TN}}{\text{TP}+\text{TN}+\text{FP}+\text{FN}},\\ \text{Recall(proportion of TP w.r.t. all positive samples)}\\ =\frac{\text{TP}}{\text{TP}+\text{FN}},\\ \text{Specificity(proportion of TN w.r.t. all negative samples)}=\\ \frac{\text{TN}}{\text{TN}+\text{FP}},\\ \text{Precision(proportion of TP among predicted positives)}\\ =\frac{\text{TP}}{\text{TP}+\text{FP}},\\ \text{F1(harmonic mean of precision and recall)}=\\ 2\cdot \frac{\text{Precision}\cdot \text{Recall}}{\text{Precision}+\text{Recall}},\\ \text{BalancedAcc(accuracy balanced by class proportion)}=\\ \frac{\text{Recall}+\text{Specificity}}{2}. \end{array}$$ (1)

In our work the threshold *t* is chosen on the validation set by maximizing the MCC coefficient, which provides a balanced single-score summary that incorporates TP, TN, FP, and FN, and is therefore less sensitive than accuracy to class imbalance:


$$\text{MCC}=\frac{\text{TP}\cdot \text{TN}-\text{FP}\cdot \text{FN}}{\sqrt{\left(\text{TP}+\text{FP}\right)\left(\text{TP}+\text{FN}\right)\left(\text{TN}+\text{FP}\right)\left(\text{TN}+\text{FN}\right)}}.$$

Threshold-independent metrics summarize performance across all thresholds. The ROC curve plots TPR(*t*) = Recall(*t*) versus FPR(*t*) = FP(*t*)/(FP(*t*)+TN(*t*)), and AUROC is the area under this curve. The precision–recall curve plots Precision(*t*) versus Recall(*t*), and AUPRC is its area; under strong class imbalance, AUPRC is often more informative than AUROC, with a random baseline equal to the positive prevalence $\pi =\frac{N_{+}}{N_{+}+N_{-}}$.

#### 2.5.3 Training hyper-parameters and baselines for comparison

The hyper-parameters and configurations used for training the FFNN are reported in Section 2.4.3. Moreover, training and validation loss curves were monitored over epochs to assess model convergence and to avoid potential overfitting by early stopping.

Hyperparameter selection was performed in preliminary experiments on a reduced subset of the training/validation interaction pairs using a grid-search strategy. We varied the number of hidden layers in {2, 4, 6}, the dropout rate in {0.1, 0.2}, and the batch size in {16, 512, 1, 024}. For each configuration, models were trained using the same optimization settings described in Section 2.4.3, and the final model was selected as the configuration that maximized validation AUPRC. No hyperparameters were tuned on the test set.

Besides the random classifier, whose expected performance are AUROC = 0.5 and AUPRC = 0.047, we employed the IntaRNA method (Mann et al., 2017) as a baseline for comparison. IntaRNA estimates interaction energy. While interaction energy can be thresholded to obtain binary predictions, which enable the computation of accuracy, balanced accuracy, precision, recall, and F1 scores, we opted to limit the comparison to AUROC and AUPRC. These metrics provide a more robust and threshold-independent evaluation of predictive performance, ensuring a fair comparison across models.

## 3 Results

We assessed the contribution of the data-augmentation strategy and the pooling operation used to obtain molecule-level embeddings. Table 1 summarizes AUROC and AUPRC results across all configurations, including a baseline random classifier, IntaRNA and *CUPID* models. For *CUPID* we compared results obtained with (Data-aug) and without (No-Data-aug) data augmentation, considering different pooling techniques, i.e., concatenation (concat), maximum (Max), and average (Avg) pooling.

**Table 1:** Comparison of AUROC and AUPRC across different experimental settings. Random baseline refers to the expected performance of the random classifiers.

**Methods**	**AUROC**	**AUPRC**
Random baseline	0.5	0.047
IntaRNA	0.544	0.055
***CUPID***		
No-Data-aug	0.658	0.078
Data-aug-Max	0.810	0.147
Data-aug-concat	0.862	0.222
Data-aug-Avg	**0.919**	**0.364**

*CUPID* models are sorted in increasing order of both AUROC and AUPRC. Bold values denote the best-performing model.

### 3.1 Random baselines

With a random classifier we can expect an AUROC = 0.5, while the estimated baseline AUPRC is:


$$\text{Baseline AUPRC}=\frac{N_{+}}{N_{+}+N_{-}}$$

where *N*_+_ is the number of positive samples, and *N*_−_ is the number of negative samples. Given the 1:20 ratio of positive to negative samples, the AUPRC baseline in the performed experiments is:


$$\text{Baseline AUPRC}=\frac{1}{1+20}\approx 0.0476.$$

Our top-performing model achieves an AUPRC of 0.364, corresponding to a *7.65-fold improvement* (0.364/0.0476). This margin quantifies the difficulty of the task: the extreme class imbalance renders precision–recall a stringent metric, and the observed gains indicate that the model extracts interaction-relevant information that is well above chance expectations.

### 3.2 IntaRNA results

**Figure 6:**
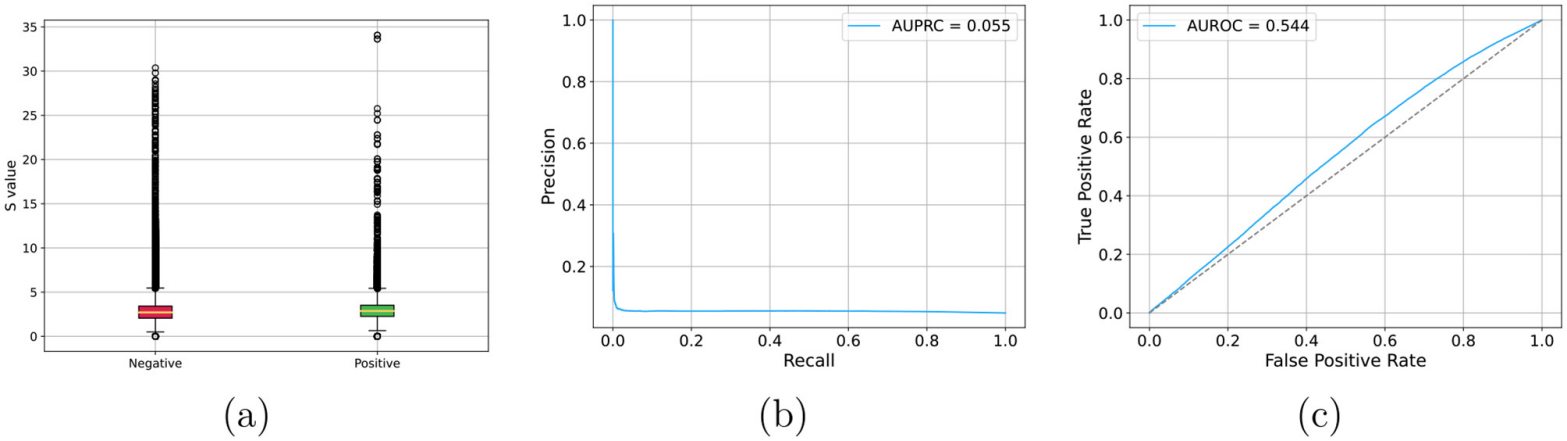
Results for IntaRNA results with augmented test set. **(a)** Distribution of predicted probabilities for negative and positive interactions; **(b)** AUPRC; **(c)** AUROC.

Figure 6 reports IntaRNA performance on the augmented test set. In this setting, IntaRNA shows limited predictive power. Its scoring function relies on thermodynamic and accessibility components (e.g., hybridization energy and site accessibility), and in our experiments we used the default parameterization. Given the heterogeneity of ncRNA classes and sequence lengths in our benchmark, improved performance would likely require careful, class-specific calibration of both energy- and accessibility-related settings. Moreover, while IntaRNA is a general thermodynamics- and accessibility-based framework and is not inherently tied to a specific organism, it was originally introduced and most extensively evaluated in bacterial sRNA–mRNA interaction settings; consequently, when applied to heterogeneous ncRNA–ncRNA interactions (including long lncRNAs and diverse eukaryotic classes), its default parameterization may be suboptimal without additional tuning.

### 3.3 *CUPID* results

**Figure 7:**
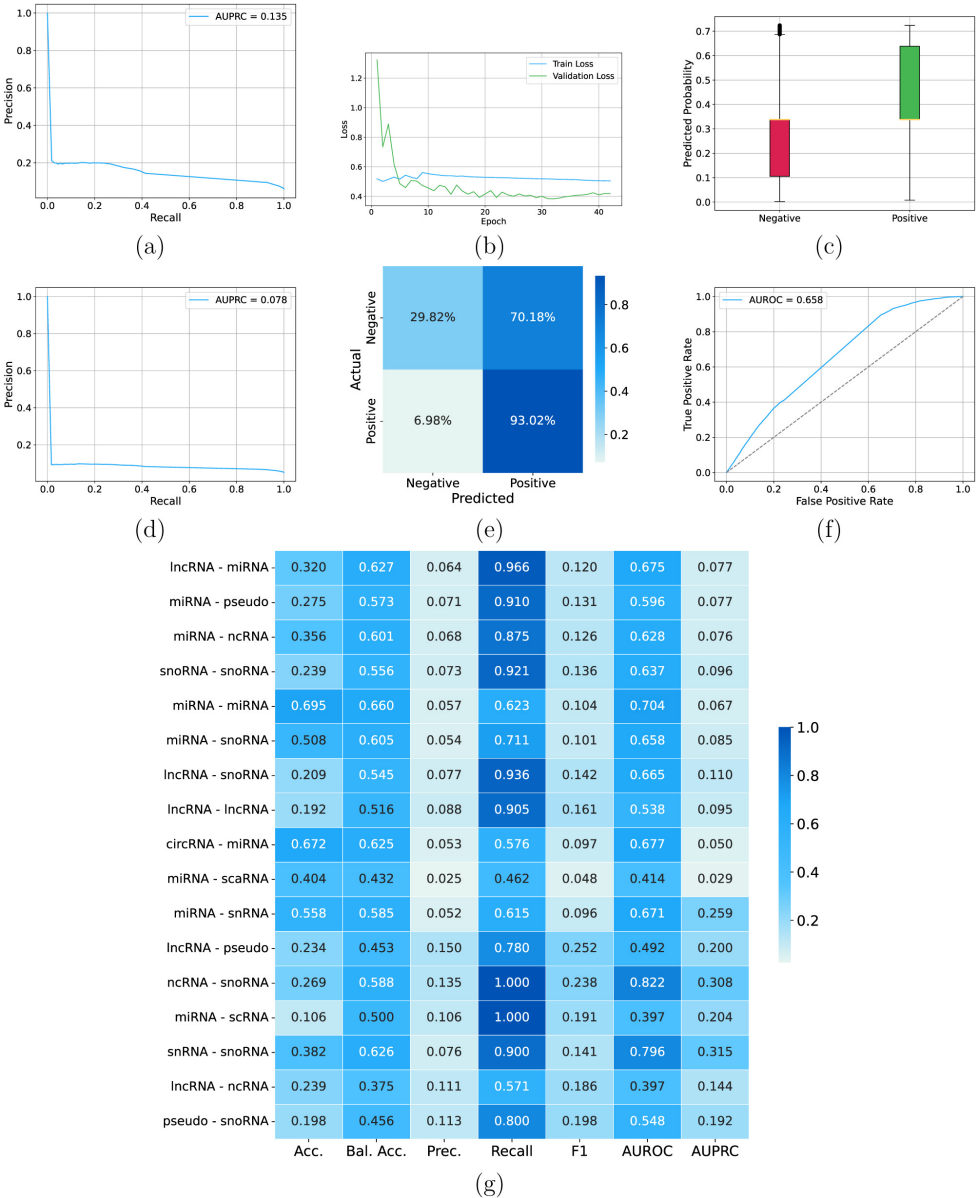
*CUPID* results with concatenated pooling, and without using augmented data. **(a)** Overall precision recall curve on the validation set including all the type of ncRNA interactions; **(b)** training and validation loss across epochs; **(c)** distribution of the *CUPID* predicted probabilities on negative and positive examples on the test set; **(d)** overall precision recall curve on the test set including all the type of ncRNA interactions; **(e)** confusion matrix on the test set; **(f)** ROC curve on the test set including all the type of ncRNA interactions; **(g)** *CUPID* results on the test set across different types on ncRNA interactions (rows) for different types of metrics (columns).

Table 1 compares all *CUPID* configurations. We first evaluated a *CUPID* model without augmentation, using concatenation of average and max pooling. Figure 7 shows the results obtained without data augmentation and with concatenated average-max pooling. The overall AUPRC results on the test set are relatively low (Figure 7c), even if a certain learning is witnessed by the AUROC largely above 0.5 (Figure 7f), and by the distribution of the predicted interaction probabilities for negative and positive examples (Figure 7c), with probabilities for positives relatively higher with respect to negatives. Nevertheless, the relatively flat trend of the training loss reveals a certain difficulty of the model to learn the data. This is reflected also in the confusion matrix where most of negative examples (70%) are misclassified (Figure 7e) and in the degradation of the AUPRC performance between validation (Figure 7a) and test (Figure 7d) data. By looking at specific ncRNA interactions, for certain interaction types (e.g., snRNA-snoRNA) we obtained good results across the different metrics, but for several ncRNA interactions (e.g., miRNA-lncRNA, miRNA-miRNA, and lncRNA-snoRNA) we achieved poor results, with AUPRC below 0.1 (Figure 7g). Summarizing Figure 7, shows that with this setting *CUPID* can provide a certain discrimination between positive and negative interactions (Figure 7c), but its precision–recall and ROC curves indicate a limited separation between positive and negative examples (Figures 7d, f).

**Figure 8:**
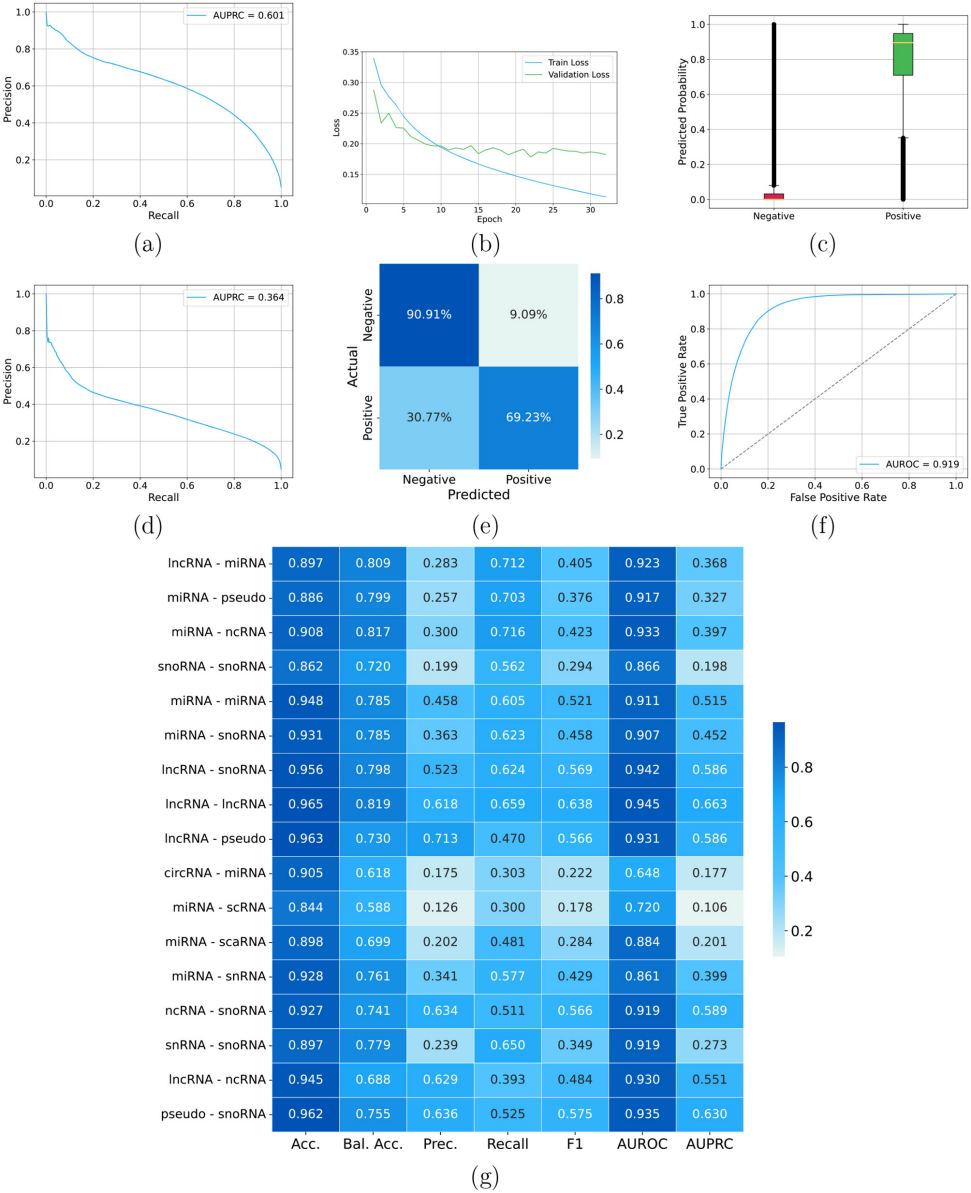
*CUPID* results with average pooling and using augmented data. **(a)** Overall precision recall curve on the validation set including all the type of ncRNA interactions; **(b)** training and validation loss across epochs; **(c)** distribution of the *CUPID* predicted probabilities on negative and positive examples on the test set; **(d)** overall precision recall curve on the test set including all the type of ncRNA interactions; **(e)** confusion matrix on the test set; **(f)** ROC curve on the test set including all the type of ncRNA interactions; **(g)** *CUPID* results on the test set across different types on ncRNA interactions (rows) for different types of metrics (columns).

Introducing data augmentation consistently improves performance (Table 1). Figure 8 shows the results obtained with data augmentation and average pooling. The AUPRC is more than 4 times larger than without data augmentation (Figure 8d and Table 1). Enlarging the size of training data by data augmentation allows the model to better learn the training data, as witnessed by the training loss that continues to decrease across epochs (Figure 8b). This results in a clear separation between the scores predicted for positive and negative examples—note that the probabilities predicted for negatives are compressed toward zero while for most positives are largely above 0.7 (even if with several outliers for both positive and negative examples, Figure 8b). The confusion matrix also confirms that the model with augmented data can better predict negative examples (Figure 8e); AUPRC (Figure 8d) significantly improves, and AUROC is larger than 0.9 (Figure 8f). Analyzing results for each specific ncRNA interaction, we can observe a significant improvement across all the considered metrics, with AUROC in most cases larger than 0.9, except for circRNA-miRNA, miRNA-scRNA, miRNA-snRNA, and miRNA-scaRNA (even if for these two last ncRNA interactions values are close to 0.9 (Figure 8g).

These results confirm that data augmentation is crucial to improve results for two main reasons: at first the model has training data enough to better generalize; second, improves generalization leveraging molecule order and orientation, two symmetries that are not guaranteed to be learned from limited training data. Augmentation effectively enforces these invariances, reducing overfitting to sequence presentation and mitigating the scarcity of positive examples.

Pooling strategy has a direct impact on the stability of the molecule-level embedding. Average pooling–yielding a smoothed representation over the full sequence—achieves the highest AUROC and AUPRC (Figure 8) compared to max pooling (Supplementary Figure S2) and concatenation pooling (Supplementary Figure S3). This indicates that interaction-relevant information is not confined to a small set of token embeddings but arises from distributed features along the sequence. Max pooling, in contrast, appears sensitive to local outliers and overly compresses positional variability, while concatenation does not provide additional benefits once augmentation is introduced. The results suggest that, for ncRNA interaction prediction, the aggregate signal across nucleotides is more informative than isolated high-activation sites.

## 4 Discussion

The results shown in this work demonstrate that RNA sequence-only inference can recover interaction signals across diverse ncRNA classes. The best-performing configuration reaches AUROC values above 0.9 on the test set, despite operating without structural, evolutionary, or thermodynamic information. This suggests that pretrained RNA language models encode latent features associated with intermolecular recognition. These features may reflect statistical regularities of pairing propensities and local compositional biases captured during pretraining, even in the absence of explicit structural supervision.

From a methodological standpoint, two contributions appear essential. First, the augmentation scheme addresses symmetries inherent to the problem. Because interacting RNAs can be presented in either order, and because sequence orientation can vary, enforcing invariance to these transformations is critical for robust generalization. Data augmentation also increases the number of examples available for training, thus improving the generalization performance of the model. Second, average pooling provides stable embeddings for ncRNA sequences. For molecules such as lncRNAs—whose functional elements are dispersed and whose lengths vary over orders of magnitude—summarizing the full sequence avoids overemphasis on isolated positions and instead captures global contextual tendencies. Moreover, to our knowledge, *CUPID* is the first model able to predict a large set of ncRNA interactions, and in principle can be applied to predict any ncRNA interaction.

The limitations observed for IntaRNA highlight the difference between energy-based and representation-based approaches. Thermodynamic models rely on explicit structural motifs and accessibility assumptions, which may not generalize to long, structured, or poorly conserved ncRNAs. In contrast, *CUPID* does not attempt to reconstruct secondary structure but leverages contextual sequence statistics learned from large corpora. These complementary perspectives suggest potential synergies: coupling language-model embeddings with coarse structural predictions could refine the discrimination between spurious and functionally relevant pairing events.

Despite these promising results, we note that the resources used to train *CUPID* are limited in size and exhibits a strong imbalance across interaction types. Although our type-constrained negative sampling preserves the empirical distribution of interaction types, rare types remain challenging; they can yield higher-variance estimates and may prevent the model from learning robust type-specific patterns. Accordingly, we emphasize AUPRC in our per-type analyses, as it is generally more informative than AUROC under severe class imbalance. Future work will benefit from larger and more balanced interaction resources, and could further improve stability on underrepresented classes via targeted strategies such as class-aware reweighting, resampling, or cost-sensitive objectives.

As larger ncRNA catalogs become available through resources such as RNAcentral (Sweeney et al., 2020), and as experimental protocols expand the coverage of ncRNA–ncRNA interactions, the training regime of models like *CUPID* can be scaled accordingly. Future developments may integrate longer receptive fields, explicit cross-attention between molecules, or joint fine-tuning on experimentally resolved interactomes. These extensions could help reveal constraints underlying ncRNA recognition and improve the resolution of regulatory maps in eukaryotic transcriptomes.

In addition, while our study focuses on a resource-efficient paradigm that leverages pre-trained RNA language models with a lightweight interaction-specific prediction head, it would be interesting to complement our analysis with baselines that train a long-context Transformer from scratch. We did not include such a baseline here because, under the current supervision regime (approximately 10^5^ interaction pairs after filtering), end-to-end training from random initialization may be difficult to optimize and may not yield generalizable representations. As larger and more diverse labeled interaction resources become available, systematic comparisons between pre-trained and from-scratch Transformer encoders will become increasingly informative.

A similar consideration holds when considering studies substituting RNA-LM models with several Transformer-based nucleotide language models. While these models could, in principle, be considered as alternative backbones for RNA sequence embeddings [e.g., models pre-trained predominantly on DNA such as Nucleotide Transformer, which has been reported to transfer RNA-related signals (Dalla-Torre et al., 2025)], we selected GenerRNA because it is pre-trained specifically on RNA sequences, provides a long-context representation and it is expected to better capture RNA-class-specific features. We therefore expect RNA-specialized pre-training to yield representations that are more directly tailored to RNA sequence regularities than more generic DNA-pre-trained alternatives, even when the latter can capture some RNA features. In this work, we focused on characterizing the proposed interaction-prediction pipeline using a single RNA-specialized backbone, including ablations on augmentation and pooling. As larger and more diverse interaction resources become available, it will be important to benchmark GenerRNA in a zero-shot setting against more general nucleotide Transformers, and to evaluate both backbones also after task-specific fine-tuning.

In summary, the results show that *CUPID* provides a scalable sequence-based framework for ncRNA–ncRNA interaction prediction, achieving AUROC larger than 0.9 for several types on ncRNA interactions. Its performance, robustness to class heterogeneity, and limited dependence on domain-specific priors make it suitable for large-scale *in silico* screening and for guiding targeted experimental profiling of ncRNA regulatory networks.

## Data Availability

Publicly available datasets were analyzed in this study. The data, the CUPID code, and the scripts to reproduce the experiments and tutorials are available from GitHub: https://github.com/AnacletoLAB/ncRNA-CUPID.

## Footnotes

1 Retrieval of interacting pairs and corresponding sequences was performed using the scripts available from the RNA-KG web site: https://github.com/AnacletoLAB/RNA-KG.

2 In RNAinter, the term “pseudo” specifically denotes RNA sequences transcribed from pseudogenes. In this context, these are transcripts derived from genes that have lost their protein–coding capability due to accumulated mutations, yet they are still produced as RNA. Similar to other ncRNAs, such pseudogene RNAs can sometimes participate in regulatory networks by, for example, acting as miRNA decoys or sponges, despite not encoding functional proteins.

## References

[B1] Aleksander S. A. Balhoff J. Carbon S. Cherry J. M. Drabkin H. J. Ebert D. . (2023). The gene ontology knowledgebase in 2023. Genetics 224:iyad031. doi: 10.1093/genetics/iyad031 36866529 PMC10158837

[B2] Ali S. A. Peffers M. J. Ormseth M. J. Jurisica I. Kapoor M. (2021). The non-coding RNA interactome in joint health and disease. Nat. Rev. Rheumatol. 17, 692–705. doi: 10.1038/s41584-021-00687-y 34588660

[B3] Alipanahi B. Delong A. Weirauch M. T. Frey B. J. (2015). Predicting the sequence specificities of DNA-and RNA-binding proteins by deep learning. Nat. Biotechnol. 33, 831–838. doi: 10.1038/nbt.3300 26213851

[B4] Cavalleri E. Cabri A. Soto-Gomez M. Bonfitto S. Perlasca P. Gliozzo J. . (2024). An ontology-based knowledge graph for representing interactions involving RNA molecules. Sci. Data 11:906. doi: 10.1038/s41597-024-03673-7 39174566 PMC11341713

[B5] Cetin S. Sefer E. (2025). A graphlet-based explanation generator for graph neural networks over biological datasets. Curr. Bioinform. 20, 840–851. doi: 10.2174/0115748936355418250114104026

[B6] Chen J. Hu Z. Sun S. Tan Q. Wang Y. Yu Q. . (2022). Interpretable RNA foundation model from unannotated data for highly accurate RNA structure and function predictions. arXiv preprint arXiv:2204.00300.

[B7] Dalla-Torre H. Gonzalez L. Mendoza-Revilla J. Lopez Carranza N. Grzywaczewski A. H. Oteri F. . (2025). Nucleotide Transformer: building and evaluating robust foundation models for human genomics. Nat. Methods 22, 287–297. doi: 10.1038/s41592-024-02523-z 39609566 PMC11810778

[B8] Engreitz J. M. Sirokman K. McDonel P. Shishkin A. A. Surka C. Russell P. . (2014). RNA-RNA interactions enable specific targeting of noncoding RNAs to nascent pre-mRNAs and chromatin sites. Cell 159, 188–199. doi: 10.1016/j.cell.2014.08.018 25259926 PMC4177037

[B9] Fabbri M. Girnita L. Varani G. Calin G. A. (2019). Decrypting noncoding RNA interactions, structures, and functional networks. Genome Res. 29, 1377–1388. doi: 10.1101/gr.247239.118 31434680 PMC6724670

[B10] Gargano M. A. Matentzoglu N. Coleman B. Addo-Lartey E. B. Anagnostopoulos A. V. Anderton J. . (2024). The Human Phenotype Ontology in 2024: phenotypes around the world. Nucleic Acids Res. 52, D1333–D1346. doi: 10.1093/nar/gkad1005 37953324 PMC10767975

[B11] Gliozzo J. Soto Gomez M. A. Bonometti A. Patak A. Casiraghi E. Valentini G. (2025). miss-SNF: a multimodal patient similarity network integration approach to handle completely missing data sources. Bioinformatics 41:btaf150. doi: 10.1101/2025.02.24.639805 40184204 PMC12011365

[B12] Li Z. Li K. Lian X. Li J. (2025). LncRNA-miRNA interaction prediction based on multi-source heterogeneous graph neural network and multi-level attention mechanism. Int. J. Biol. Macromol. 319:145614. doi: 10.1016/j.ijbiomac.2025.145614 40582680

[B13] Lorenzi L. Chiu H. S. Avila Cobos F. Gross S. Volders P. J. Cannoodt R. . (2021). The RNA atlas expands the catalog of human non-coding RNAs. Nat. Biotechnol. 39, 1453–1465. doi: 10.1038/s41587-021-00936-1 34140680

[B14] Mann M. Wright P. R. Backofen R. (2017). IntaRNA 2.0: enhanced and customizable prediction of RNA-RNA interactions. Nucleic Acids Res. 45, W435–W439. doi: 10.1093/nar/gkx279 28472523 PMC5570192

[B15] Matthews B. W. (1975). Comparison of the predicted and observed secondary structure of t4 phage lysozyme. Biochim. Biophys. Acta 405, 442–451. doi: 10.1016/0005-2795(75)90109-9 1180967

[B16] Nicolini M. Saitto E. Jimenez-Franco R. Cavalleri E. Alfonso A. J. G. Malchiodi D. . (2025a). Fine-tuning of conditional Transformers improves in silico enzyme prediction and generation. Comput. Struct. Biotechnol. J. 27, 1318–1334. doi: 10.1016/j.csbj.2025.03.037 40235640 PMC11999079

[B17] Nicolini M. Stacchietti F. Cano C. Casiraghi E. Valentini G. (2025b). “A transformer-based model to predict micro RNA interactions,” in 18th InteRNAtional Work-Conference on Artificial Neural Networks, IWANN 2025. doi: 10.1007/978-3-032-02725-2_8

[B18] Radford A. Wu J. Child R. Luan D. Amodei D. Sutskever I. . (2019). Language models are unsupervised multitask learners. OpenAI Blog 1:9.

[B19] Sapoval N. Aghazadeh A. Nute M. G. Antunes D. A. Balaji A. Baraniuk R. . (2022). Current progress and open challenges for applying deep learning across the biosciences. Nat. Commun. 13:1728. doi: 10.1038/s41467-022-29268-7 35365602 PMC8976012

[B20] Sefer E. (2025). Drgat: predicting drug responses via diffusion-based graph attention network. J. Comput. Biol. 32, 330–350. doi: 10.1089/cmb.2024.0807 39639802

[B21] Sennrich R. Haddow B. Birch A. (2016). “Neural machine translation of rare words with subword units,” in Proceedings of the 54th Annual Meeting of the Association for Computational Linguistics (Berlin, Germany: Association for Computational Linguistics), 1715–1725. doi: 10.18653/v1/P16-1162

[B22] Shen T. Hu Z. Sun S. Liu D. Wong F. Wang J. . (2024). Accurate RNA 3D structure prediction using a language model-based deep learning approach. Nat. Methods 21, 2287–2298. doi: 10.1038/s41592-024-02487-0 39572716 PMC11621015

[B23] Sun S.-L. Jiang Y.-Y. Yang J.-P. Xiu Y.-H. Bilal A. Long H.-X. (2025). Predicting noncoding RNA and disease associations using multigraph contrastive learning. Sci. Rep. 15:230. doi: 10.1038/s41598-024-81862-5 39747154 PMC11695719

[B24] Sweeney B. A. Petrov A. I. Ribas C. E. Finn R. D. Bateman A. Szymanski M. . (2020). RNAcentral 2021: secondary structure integration, improved sequence search and new member databases. Nucleic Acids Res. 49, D212–D220. doi: 10.1093/nar/gkaa921 33106848 PMC7779037

[B25] Tian X. Shen L. Wang Z. Zhou L. Peng L. (2021). A novel lncRNA-protein interaction prediction method based on deep forest with cascade forest structure. Sci. Rep. 11:18881. doi: 10.1038/s41598-021-98277-1 34556758 PMC8460650

[B26] Torgano F. Soto Gomez M. Zignani M. Gliozzo J. Cavalleri E. Mesiti M. . (2025). RNA knowledge-graph analysis through homogeneous embedding methods. Bioinform. Adv. 5:vbaf109. doi: 10.1101/2025.02.17.638592 40496493 PMC12150776

[B27] Umu S. U. Gardner P. P. (2017). A comprehensive benchmark of RNA-RNA interaction prediction tools for all domains of life. Bioinformatics 33, 988–996. doi: 10.1093/bioinformatics/btw728 27993777 PMC5408919

[B28] Valentini G. Malchiodi D. Gliozzo J. Mesiti M. Soto-Gomez M. Cabri A. . (2023). The promises of large language models for protein design and modeling. Front. Bioinform. 3:1304099. doi: 10.3389/fbinf.2023.1304099 38076030 PMC10701588

[B29] Vasilevsky N. A. Toro S. Matentzoglu N. Flack J. E. Mullen K. R. Hegde H. . (2025). Mondo: integrating disease terminology across communities. Genetics 2025:iyaf215. doi: 10.1093/genetics/iyaf215 41052288 PMC13050200

[B30] Wang W. Zhang L. Sun J. Zhao Q. Shuai J. (2022). Predicting the potential human lncRNA–miRNA interactions based on graph convolution network with conditional random field. Brief. Bioinform. 23:bbac463. doi: 10.1093/bib/bbac463 36305458

[B31] Wei J. Chen S. Zong L. Gao X. Li Y. (2022). Protein–RNA interaction prediction with deep learning: structure matters. Brief. Bioinform. 23:bbab540. doi: 10.1093/bib/bbab540 34929730 PMC8790951

[B32] Yang T. He Y. Wang Y. (2025). Introducing tec-lncmir for prediction of lncRNA-miRNA interactions through deep learning of RNA sequences. Brief. Bioinform. 26:bbaf046. doi: 10.1093/bib/bbaf046 39927859 PMC11808807

[B33] Yu H. Yang H. Sun W. Yan Z. Yang X. Zhang H. . (2024). An interpretable RNA foundation model for exploring functional RNA motifs in plants. Nat. Mach. Intell. 6, 1616–1625. doi: 10.1038/s42256-024-00946-z 39703563 PMC11652376

[B34] Yu X. Jiang L. Jin S. Zeng X. Liu X. (2022). premLI: a pre-trained method to uncover microRNA–lncRNA potential interactions. Brief. Bioinform. 23:bbab470. doi: 10.1093/bib/bbab470 34850810

[B35] Zhao Y. Oono K. Takizawa H. Kotera M. (2024). GenerRNA: a generative pre-trained language model for de novo RNA design. PLoS ONE 19:e0310814. doi: 10.1371/jouRNAl.pone.0310814 39352899 PMC11444397

