# Supplementary material for: Computational understanding of non-coding RNA pairwise interactions
Source: Front Artif Intell. 2026 Feb 18;9:1749205. doi: 10.3389/frai.2026.1749205 (PMC12957212; doi:10.3389/frai.2026.1749205)
Supplement: Supplementary file 1 [file Data_Sheet_1.pdf]

frontiers

Computational Understanding of non-coding RNA Pairwise Interactions - Supplementary Material

S1 SUPPLEMENTARY FIGURES

	lncRNA	miRNA	ncRNA	pseudo	scRNA	scaRNA	snRNA	snoRNA
circRNA	-	1180	-	-	-	-	-	-
lncRNA	5340	219092	504	1480	-	-	-	6140
miRNA	-	7456	23256	67412	728	460	444	7112
ncRNA	-	-	-	-	-	-	-	784
pseudo	-	-	-	-	-	-	-	372
snRNA	-	-	-	-	-	-	-	376
snoRNA	-	-	-	-	-	-	-	17288

Figure S1: Training set ncRNA interactions distribution after data augmentation. Numbers represent the cardinality of the interactions.

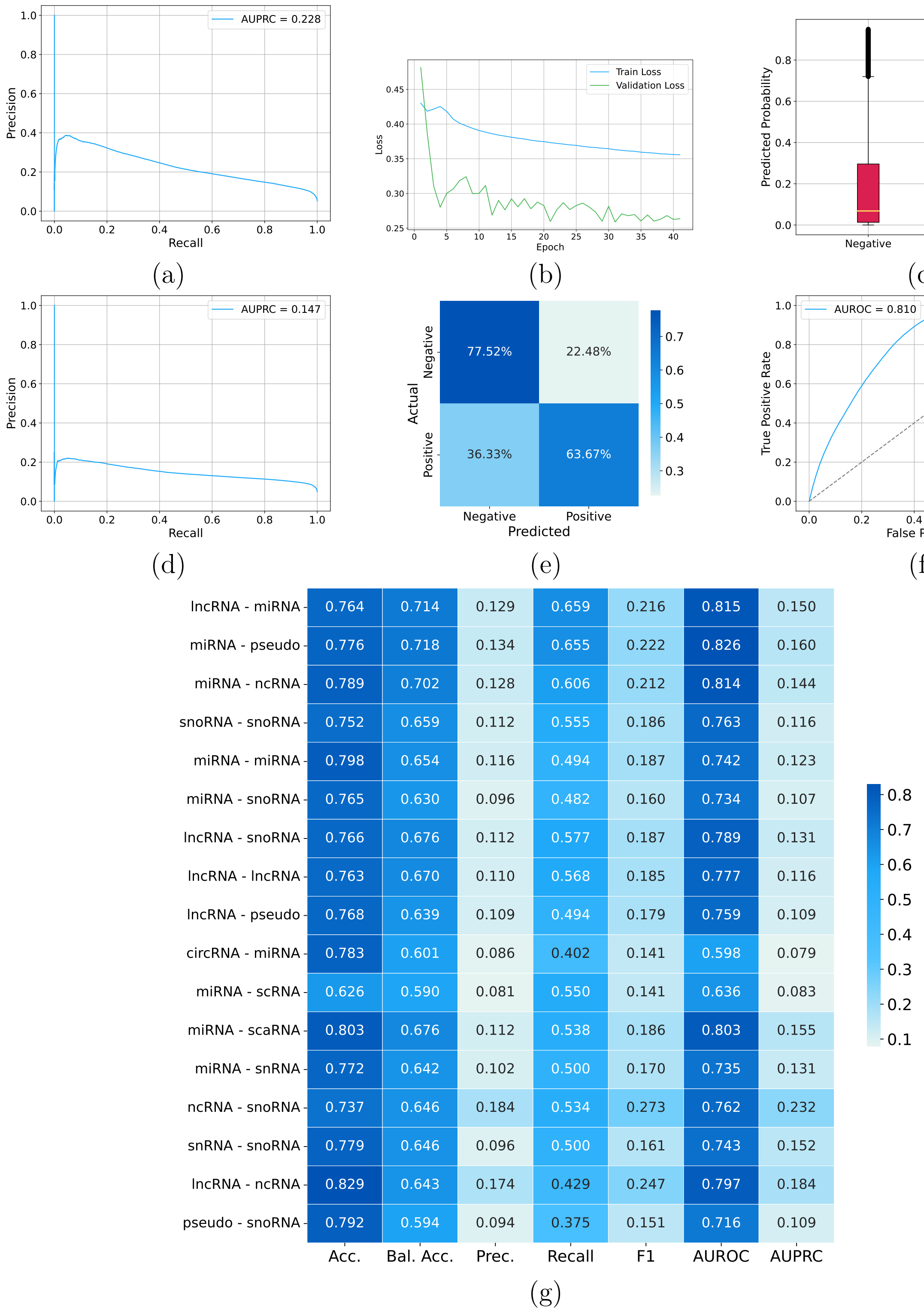

Figure S2: *CUPID* results with Max pooling, and using augmented data. (a) Overall precision recall curve on the validation set including all the type of ncRNA interactions; (b) Training and validation loss across epochs; (c) Distribution of the *CUPID* predicted probabilities on negative and positive examples on the test set; (d) Overall precision recall curve on the test set including all the type of ncRNA interactions; (e) Confusion matrix on the test set; (f) ROC curve on the test set including all the type of ncRNA interactions; (g) *CUPID* results on the test set across different types on ncRNA interactions (rows) for different types of metrics (columns).

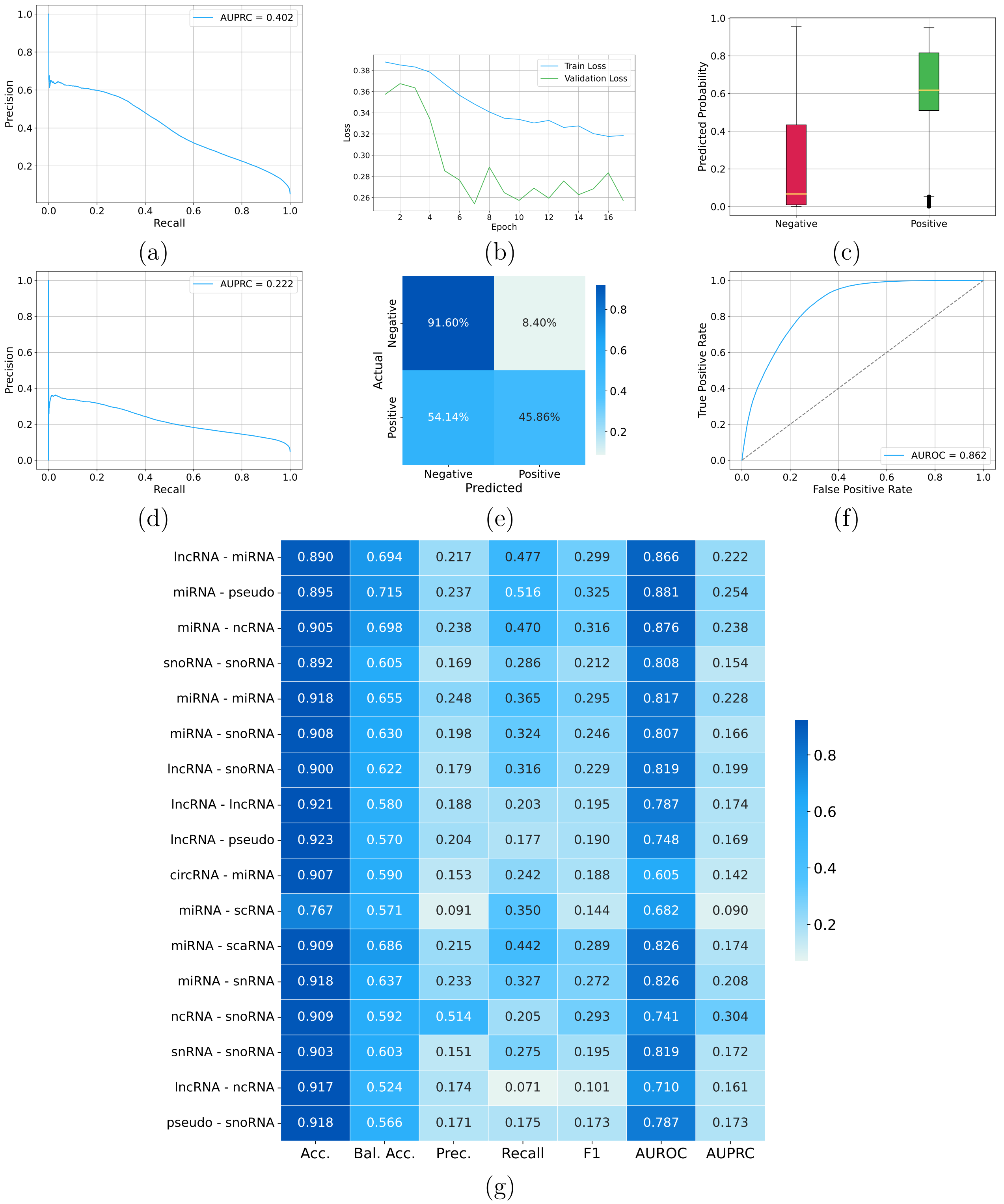

Figure S3: *CUPID* results with concatenation of average and maximum pooling, and using augmented data. (a) Overall precision recall curve on the validation set including all the type of ncRNA interactions; (b) Training and validation loss across epochs; (c) Distribution of the *CUPID* predicted probabilities on negative and positive examples on the test set; (d) Overall precision recall curve on the test set including all the type of ncRNA interactions; (e) Confusion matrix on the test set; (f) ROC curve on the test set including all the type of ncRNA interactions; (g) *CUPID* results on the test set across different types on ncRNA interactions (rows) for different types of metrics (columns).
